# Disability and COVID-19: ensuring no one is left behind

**DOI:** 10.1186/s13690-021-00656-7

**Published:** 2021-08-20

**Authors:** Consortium “United Nations workstream on COVID-19 disability inclusive health response and recovery”, Alarcos Cieza, Kaloyan Kamenov, Ola Abu Al Ghaib, Alessandra Aresu, Somnath Chatterji, Facundo Chavez, Jarrod Clyne, Nathalie Drew, Michelle Funk, Andrea Guzman, Eleonora Guzzi, Chapal Khasnabis, Bente Mikkelsen, Ren Minghui, Gopal Mitra, Priyanka Narahari, Gisela Nauk, Alice Priddy, Alaa Sabeh, Maria Soledad Cisternas Reyes, Javier Vasquez, Roxana Widmer-Iliescu

**Affiliations:** 1 World Health Organization, Geneva, Switzerland; 2 UN Partnership on Persons with Disabilities Fund, United Nations Development Program, New York, USA; 3 International Disability and Development Consortium, Brussels, Belgium; 4 Office of the United Nations High Commissioner for Human Rights, Geneva, Switzerland; 5 International Disability Alliance, Geneva, Switzerland; 6 Executive Office of the Secretary-General, United Nations, New York, USA; 7 United Nations Entity for Gender Equality and the Empowerment of Women, New York, USA; 8 Economic and Social Commission for Western Asia, Beirut, Lebanon; 9 Special Envoy of the United Nations Secretary General on Disability and Accessibility, New York, USA; 10 International Telecommunication Union, Geneva, Switzerland

**Keywords:** Covid-19, Disability, Health systems

## Abstract

The United Nations’ Sustainable Development Agenda calls for targeted attention to the needs and rights of the most vulnerable populations to ensure a life of dignity and human security for all. In this paper, we argue that persons with disabilities are in a disproportionately vulnerable situation in public health emergencies. By using the example of Coronavirus disease 2019 (Covid-19), we explain why that is and call for the systematic consideration of the needs and rights of persons with disabilities during the response to the outbreak and during the recovery phase. Otherwise, equity will continue to be merely an aspiration during this COVID-19 emergency - as it will in future health emergencies.

**Supplementary Information:**

The online version contains supplementary material available at 10.1186/s13690-021-00656-7.

## Main text

Persons with disabilities are a heterogeneous group in terms of identity, gender, age, and underlying health conditions or impairments, and their diversity leads to different health-care needs. For example, children with underlying conditions such as cerebral palsy or muscular dystrophy require early identification and support to allow them to optimize their development and functioning [1]. In their contact with health care services, people with severe vision or hearing impairments require public health information and interventions that are accommodated and accessible [2, 3]. People with chronic conditions associated with high levels of disability, such as spinal cord injury, stroke, rheumatoid arthritis or chronic obstructive pulmonary disease often require long-term care delivered by specialized health professionals who need to be trained to recognize the specific needs of each person with disability [4–7]. Older persons are likely to experience more impairments associated with frailty that often results in elevated health-care use and need for caregiver support [8]. Alongside their underlying health conditions and impairments, the environment in which people live will define their experience of disability. Attitudinal and environmental barriers such as inaccessible education, transportation, employment and health care, hinder persons with disabilities to fully and effectively participate in society on an equal basis with others.

Regardless of the diverse nature of the disability, three increased risks are commonly experienced by persons with disabilities during the COVID-19 pandemic: risk in contracting COVID-19, in developing severe symptoms of COVID-19 or dying from the complications of the disease, and of having poorer health during and after the outbreak, whether or not they are infected with COVID-19.

Firstly, persons with disabilities are at higher risk of contracting COVID-19 virus, mainly due to environmental barriers that limit effective protection against infection. One such barrier is the lack of timely and accessible public health information that can result either in not receiving the necessary messages or receiving them too late. Few countries and public health organizations distribute guidelines in formats accessible to persons with disabilities – formats including the use of plain language, Easyread formats, electronic screen readers, or captioning and sign language. Also, current public health messaging often does not reach people who are institutionalized or those with mental health and substance use disorders who have limited personal resources, unstable housing conditions, or are homeless [9].

Other barriers relate to the lack of accommodation necessary to facilitate the implementation of basic public health actions, such as frequent handwashing and maintaining physical distance [10]. During the COVID-19 outbreak, locations of superspreading events such as long-term care facilities have been reported in many countries. It has been made clear that in institutional settings where physical distancing is challenging, persons with disabilities are more prone to contracting the disease [11]. This applies also to older persons being cared for in nursing homes or similar facilities where unregulated and poor-quality services, including inadequate infection prevention and control, in some, have led to the spread of the virus and increased mortality [12, 13]. In fact, data from 31 countries identified through a comprehensive review of available evidence, revealed that the deaths occurring in long-term care institutions as proportion of the total Covid-19 related deaths in the respective countries was extremely high. By September 23, 2020, the average percentage of mortality in institutions was 40.3% with some countries reaching as high as 85%. Supplementary Material 1 provides full details for each country.

Secondly, people with underlying health conditions associated with high levels of disability have an increased risk of developing severe symptoms of COVID-19 or dying. The evidence supporting this claim comes from studies that show that people with diabetes mellitus, cerebrovascular disease, chronic obstructive pulmonary disease, and coronary artery disease have a poorer prognosis with COVID-19 [14–18]. Individuals with inflammatory rheumatic disease are also at risk of severe infection due to their immuno-compromised state [19]. In addition, a big proportion of persons with disabilities are over 60 years of age, and as age is one of the strongest risk factors for mortality by Covid-19, persons with disabilities are at particular risk [20]. In fact, published studies show a strong age gradient in the risk of morbidity and mortality from the virus [21], and fatality rates among those over 80 years of age are five times the global average [22].

Despite the increased risks of developing severe symptoms or dying during COVID-19, persons with disabilities have been frequently denied their right to receive the same range of health care as others due to triaging practices in hospitals [23]. This can be seen mainly in overburdened health systems faced with limited supplies of resources, such as emergency care beds and ventilators [24–26]. Denial of COVID-19 treatment based on disability can be seen in many countries; in the United States, for example, the Alabama guidelines recommended that hospitals withhold ventilators from “patients with severe or profound mental retardation”, dementia, or severe traumatic brain injury, whereas Utah guidelines recommended excluding patients with advanced neuromuscular diseases who require assistance with activities of daily living [27, 28]. Such discriminatory decisions contributed to the disproportionately high mortality rates amongst persons with disabilities. In the United Kingdom, statistics showed that persons with disabilities accounted for 59% of all COVID-19 deaths in the country [29]. In addition to the denial of health care, more and more evidence for amplified aggression and coercion within the health sector towards persons with psychosocial disabilities can be seen in many countries during the pandemic, leading to severe consequences including violence and even death [30].

Thirdly, persons with disabilities have an increased risk of poorer health during and after the outbreak even without contracting COVID-19. This is because of measures some countries have relied on to control the outbreak. Country lockdowns can lead to restricted access to public spaces, making it harder to undertake healthy behaviors, such as physical activity or socializing. Social distancing during the pandemic has been harder for people with vision impairment, and the common use of face masks have affected the ability of people with hearing loss to lip read and communicate. These measures have also affected the access to medicines, health products or event food for persons with disabilities [31]. Very often restrictions have severely affected the mental health state of millions of people who have not received adequate services to address their needs [32]. Country measures have even more severe health deteriorating consequences when health is already compromised [33]. People living with dementia, for example, can feel lonely and withdrawn and their cognitive states can deteriorate since they may have little experience with telecommunication and depend primarily on in-person support [34]. Individuals with neurodevelopmental conditions such as Attention Deficit Hyperactivity Disorder (ADHD), being particularly vulnerable to the physical distancing measures, may feel increasingly distressed and anxious by the restrictions [35]. Furthermore, disruptions in the provision of social care have had a devastating impact on persons with disabilities who rely on it, and many have had to rely on the support of their families. The closure of day centers or any other means of socialization has left persons with disabilities isolated [36]. Also, children with special and educational needs and disabilities have been excluded from receiving education in some countries because of lack of accessible information and educational materials in accessible formats [31].

In addition, poorer health is associated with reducing essential health services to an absolute minimum during the COVID-19 outbreak. A person with a disability may require frequent access to health-care services due to their underlying health conditions [2]; disruption to these services puts them at a disadvantage in maintaining their general health. In China, persons with psychosocial disabilities who usually visit psychiatric outpatient clinics on a monthly basis to obtain maintenance medications have been frequently denied access due to mass quarantines and restrictions to public transport [37]. Table 1 summarizes the main risks that persons with disabilities commonly experience during the current Covid-19 pandemic, the factors and barriers associated with these risks, and the main areas where the actions in the post-pandemic era need to focus.

**Table 1:** Risks experienced by persons with disabilities in the Covid-19 pandemic, barriers associated with the risks and key response areas for the post-pandemic era

Risks	Factors associated with the risks	Response
Contracting Covid-19	Societal: - Lack of timely and accessible public health information - Lack of accommodation to facilitate the implementation of basic public health actions - Lack of effective protection measures in institutionalized care	- Access to health services which includes removing any communication, physical and attitudinal barriers - Disability-inclusive national emergency preparedness and response plans - Disability-inclusive cross-sectorial public health interventions that target various determinants of health
Developing severe symptoms or dying	Biomedical: - Preexisting health condition and comorbidities Societal: - Denied healthcare because of triaging practice	
Poorer health during and after pandemic	Societal: - Measures to control the outbreak without accommodation - Exacerbated impact of lack of physical activity and social contacts - Reduction of essential health services and social care	

To respond to these challenges, targeted actions to mitigate the risks and secure the rights of persons with disabilities, including the rights to life and to the highest attainable standard of health during the COVID-19 outbreak have already been proposed in various documents, including recent guidance from the United Nations Secretary General’s office and the World Health Organization [38–43]. Those actions need urgently to be put into practice.

Governments, the public health community, all professionals providing COVID-19-related care and general health care, as well as civil society need to look beyond the current public health crisis and consider now how to build back better our health systems so that they are more inclusive for everyone - including persons with disabilities. The health sector response needs to target three main areas - access to health services, disability-inclusive national emergency preparedness and response plans, and disability-inclusive cross-sectorial public health interventions. The long-term outcome of COVID-19 must be to set a new precedent by building universally designed health systems, inclusive economies, systems rooted in the communities, reaching out and empowering persons with disabilities and guaranteeing equal opportunities for all [44]. This can only be achieved if the Sustainable development Goals (SDGs) in general and SDG 3 in particular include targeted disability-inclusive measures in line with the UN Convention on the Rights of Persons with Disabilities.

For the implementation of SDG 3 there is a proposed Global Action Plan with seven key accelerators of progress [45]. All these accelerators need to be disability-inclusive, if we want to ensure that persons with disabilities are not left behind by the health sector, especially during future health emergencies. These are some actions that need to be included:

### Universally accessible primary health care

Free or affordable, effective and sustainable primary health care is the cornerstone to achieving the health-related SDG targets for everyone. Primary health care brings health services, facilities and goods closer to where people live and work and links those services to higher levels of care. Providing barrier-free access to primary health care is therefore a fundamental right for everyone but especially for those requiring services on a regular basis, as is the case for some persons with disabilities. In addition to the accessible primary health care, all service delivery within the health systems should be appropriately and proportionately delivered to all people on an equal basis. This includes equitable access to COVID-19 vaccination and ensuring that equity in access is a guiding principle for all immunization programmes [46].

### Sustainable and accessible financing for health with social protection programmes

Sustainable and accessible financing that reduces the unmet need for health services and the financial hardship to users from out-of-pocket payments and disproportionate economic hardship need to be complemented by social protection programmes, so that extra costs of medicines, assistive products or personal support can be covered without incurring penalties for decrements in health and functioning.

### Inclusive community and civil society engagement

Persons with disabilities and their family members or caregivers can present their experience, perspective and expertise to knowledge-generation and policymaking. However, to meaningfully participate in the design, implementation and monitoring of health systems, accessibility, non-discrimination, and support if requested, must be ensured. Adopting this approach not only achieves accountability but ensures that no one is left behind.

### Proportionate universalism in determinants of health

Determinants of health need to be addressed in such a way that everyone is included in health interventions and public health strategies. The nature, scale and intensity of the interventions must equate to the nature, scale and intensity of the specific needs of those experiencing disability so that the outcome is equal for all. This is vital for ensuring that gains are maximized for everyone equally across the SDGs, including persons with disabilities.

### Innovative programming in fragile and vulnerable settings

A disability-inclusive humanitarian response must be based on thorough analysis of the needs and factors contributing to the heightened risks faced by persons with disabilities, including age, gender and barriers they encounter in accessing health care and assistance in a humanitarian context, such as a pandemic. The strategic response includes mainstreaming interventions to ensure accessibility to all (e.g. making “Water, sanitation and hygiene (WASH)” facilities physically accessible to all) and addressing disability-related needs directly (e.g. ensuring the provision of assistive products) to ensure that persons with disabilities have access to health services on an equal basis with others [47].

### Innovation and universal access

Innovation is critical to improving the quality and efficiency of public services; sustainable and equitable access ensures better availability of services to those who need them most. It is, therefore, paramount that any newly developed or re-formed services innovate their thinking as regards inclusion and ensure that persons with disabilities have equitable access to information in accessible formats, facilities, programmes, services and goods.

### Data and digital health

Good quality, comprehensive, accessible and appropriately disaggregated data are necessary to understand the needs and current situation of persons with disabilities. Such data can show, for example, whether persons with disabilities die more often because of COVID-19, which includes identifying the barriers they face in accessing healthcare interventions and the health consequences of this. This information is key to designing inclusive programmes, policies and legislation, guiding investment and public decisions, and measuring progress. Digital technologies must be made accessible for all. During the COVID-19 outbreak, for example, information on symptom tracking, contact tracing or testing, delivered through digital technology needs to be made accessible so that disability-related data can also be collected.

## Conclusion

COVID-19 has created a human crisis on an unprecedented scale, which has disproportionately impacted persons with disabilities. Nonetheless, the pandemic provides an opportunity to build better and more equitable health sector where everyone has equal access to information, facilities, programmes, services and goods, and where nobody is left behind. Current available health services must embrace more digital technology to deliver Digitally Enhanced Healthcare with virtual or remote services to ensure Good Health and Well-being of the persons with disabilities. It is evident that the targets of SDG 3 cannot be met without accelerated progress in addressing health disparities, mitigating digital divide and reducing vulnerability across countries. Therefore, the rights and needs of persons with disabilities need to be recognized by policymakers and the entire health-care sector as being essential to achieving SDG 3 and universal health coverage.

## Supplementary Information

**Additional file 1 Table 1.** Covid-19 related mortality in long-term care institutions: results from a comprehensive review (Updated: September 23, 2020).

## Data Availability

All data generated or analysed during this study are included in this published article [and its supplementary information files].

## Abbreviations

Covid-19: Coronavirus disease 2019
ADHD: Attention Deficit Hyperactivity Disorder
SDGs: Sustainable development Goals
WASH: Water, sanitation and hygiene
