# Supplementary material for: Disability and COVID-19: ensuring no one is left behind
Source: Arch Public Health. 2021 Aug 20;79:148. doi: 10.1186/s13690-021-00656-7 (PMC8377454; doi:10.1186/s13690-021-00656-7)
Supplement: Supplementary file 1 — Additional file 1 Table 1. Covid-19 related mortality in long-term care institutions: results from a comprehensive review (Updated: September 23, 2020). [file 13690_2021_656_MOESM1_ESM.docx]

**Annex 1**

**Covid-19 related mortality in long-term care institutions: Results from a comprehensive review**

*Table 1. Number of COVID-19 related deaths that have occurred in long-term care institutions (Updated: September 23, 2020)*

| **Country** | **WHO Region** | **Date of data count** | **Number of deaths in institutions** | **% of deaths in institutions from all deaths in the country** | **Source** | **Data collected** |
| --- | --- | --- | --- | --- | --- | --- |
| Australia | Western Pacific | September 23 | 642 | 74.7% | Government of Australia (1) | People who have tested positive. |
| Austria | European | June 5 | 222 | 34% | Government of Austria (2) | People who have tested positive. |
| Belgium | European | September 23 | 4,851 | 48.7% | Sciensano - a public research institution (3) | People who have tested positive + suspected cases. |
| Canada | Americas | June 4 | 6,236 | 85% | ILTCPN (4) | People who have tested positive + suspected cases. |
| Denmark | European | September 23 | 224 | 34.9% | Statens Serum Institute (5) | People who have tested positive. |
| England | European | September 11 | 14,818 | 29.7% | Office for National Statistics (6) | People who have tested positive + suspected cases. |
| Finland | European | September 23 | 147 | 43% | Finnish Institute for Health and Welfare (7) | People who have tested positive. Data is for social welfare 24-hour units. |
| France | European | September 15 | 14,528  - 14,214 died in homes for the elderly  - 281 died in facilities for people with disabilities  - 33 died in other establishments | 46,9% | Government of France (8) | People who have tested positive + suspected cases. |
| Germany | European | September 23 | 3690 | 39,2% | Robert Koch Institute (9) | People who have tested positive. Communal establishments such as prisons have been included in the total count of institutional deaths. |
| Hong Kong | Western Pacific | July 30 | 0 | 0% | Government of Hong Kong (10) | People who have tested positive. |
| Hungary | European | June 2 | 127 | 24% | Government of Hungary (11) | People who have tested positive. |
| Ireland | European | June 22 | 1,086  (963 of which are linked to nursing homes) | 63,2% | Government of Republic of Ireland (12) | People who have tested positive + suspected cases. |
| Israel | European | June 24 | 137 | 45% | Government of Israel + ILTCPN (13, 14) | People who have tested positive. |
| Italy | European | May 5 | 3772 | 12,9% | National Health Institute (15) | People who have tested positive + suspected cases. |
| Jordan | Eastern Mediterranean | July 7 | 0 | 0% | ILTCPN (14) | People who have tested positive. |
| Malta | European | July 7 | 0 | 0% | Government of Malta + ILTCPN (14, 16) | People who have tested positive. |
| Netherlands | European | September 23 | 2884 | 45,8% | Government of The Netherlands (17) | People who have tested positive. |
| New Zealand | Western Pacific | August 3 | 16 | 72,2% | Government of New Zealand + ILTCPN (14, 18) | People who have tested positive + suspected cases. |
| Northern Ireland | European | September 23 | 433 | 48,8% | Northern Ireland Statistics and Research Agency (19) | People who have tested positive + suspected cases. |
| Norway | European | September 20 | 156 | 58% | Norwegian Institute of Public Health (20) | People who have tested positive. |
| Portugal | European | May 9 | 450 | 40% | Government of Portugal (21) | The press release does not provide details on counted cases. |
| Scotland | European | September 16 | 1,966 | 46.4% | National Records of Scotland (22) | People who have tested positive + suspected cases. |
| Singapore | Western Pacific | July 7 | 2 | 7.4% | Government of Singapore (23) | People who have tested positive. |
| Slovenia | European | May 20 | 85 | 81% | Government of Slovenia (24, 25) | People who have tested positive. |
| South Korea | Western Pacific | April 30 | 84 | 34% | ILTCPN (14) | People who have tested positive. |
| Spain | European | September 23 | 20,237 | 65.2% | Government of Spain (26, 27) | People who have tested positive + suspected cases. |
| Sweden | European | September 23 | 2,627 | 45,2% | National Board of Health and Welfare (28) | People who have tested positive + suspected cases. |
| Switzerland | European | May 19 | 1,002 | 53% | Swiss cantonal data (29) | People who have tested positive. |
| Turkey | European | May 7 | 150 | 4% | ILTCPN (14) | People who have tested positive. |
| Wales | European | September 11 | 706 | 27.5% | Office for National Statistics (6) | People who have tested positive + suspected cases. |
| USA | Americas | September 23 | 80,006 | 40% | Kaiser Family Foundation (30) | People who have tested positive. |

## Detailed country information

## Australia

The Department of Health of the Australian Government updates on a daily basis the number of deaths of people who have tested positive for COVID-19, living in Australian Government–subsidized residential aged care facilities. By September 23, there were 642 people who died in these institutions, constituting 74.7% of the overall number of COVID-19 related deaths in the country. In addition, there were also 7 deaths among people who used publicly subsidized home care. The data represents the place of residence, not the place of death, indicating that the number might also include residents who died in hospital.

## Austria

Data from the Austrian epidemiological alert system published on June 5 showed that 222 residents in care homes for older people had died with COVID-19. This number constitutes 34% of the 646 total deaths linked to COVID-19 in Austria on the same date. No updated data have been published since June 5.

## Belgium

COVID-19 related data in Belgium is collected by Sciensano, a public research institution, which publishes detailed epidemiological reports daily. They include data on the number of deaths in care homes, both “confirmed” (through a test or a chest scan), and “suspected” cases where the patient had not been tested but a doctor has confirmed symptoms consistent with COVID-19. By September 23, 4851 confirmed and suspected cases had died in care homes which constitute half (48.7%) of all COVID-19 related deaths in Belgium.

## Canada

Canada does not provide a nation-wide estimate for number of COVID-19 related deaths in institutions. Many provinces provide data in their epidemiological reports (e.g for British Columbia or Ontario), however, other provinces and territories have either no cases or too few cases in care homes and provide no meaningful estimates. The ILTCPN published a report in which available data until June 4 from different provinces were compiled and found that 6,236 people died in care homes. This amounted to 85% of all COVID-19 deaths in Canada. It has to be noted that the variation in reporting across provinces makes it hard to determine whether the location of death was in a care home for all cases. Also, some official sources have reported total counts of deaths in care homes, without defining whether COVID-19 has been determined as a contributing or underlying cause of death. This may have led to a potential inflation of the estimates of deaths due to COVID-19.

## Denmark

Statens Serum Institute in Denmark provides detailed COVID-19 epidemiological reports on a daily basis, including number of confirmed COVID-19 deaths in nursing homes. By September 23, 224 people had died in nursing homes, constituting 34.9% of all COVID-19 related deaths in Denmark.

## England

In England, the Office for National Statistics (ONS) provides weekly updates of registered deaths which differ from the figures provided by NHS in that they include all deaths where

“COVID-19” was mentioned (by a doctor) on death certificates (using ICD10 codes U07.1 and U07.2). Up to the September 11, there were 14,818 deaths that occurred in care homes in England – a 29,7% of all COVID-19 related deaths in the country. Care homes include homes for the chronic sick, nursing homes, homes for people with mental health problems and non-NHS multi-function sites. In addition, 736 deaths occurred in hospices, which include Sue Ryder Homes, Marie Curie Centres, oncology centres, voluntary hospice units, and palliative care centres. Another 209 deaths occurred in other communal establishments including schools for people with learning disabilities, holiday homes and hotels, common lodging houses, aged persons’ accommodation, assessment centres, schools, convents and monasteries, and nurses’ homes.

## Finland

The Finnish Institute for Health and Welfare publishes daily epidemiological reports on COVID-19, including information on mortality in social welfare 24-hour units. According to the report from September 23, 43% or 147 of all deaths occurred in these institutions.

## France

French government publishes weekly reports on COVID-19 including detailed information on mortality in different types of institutions. Overall, by September 15, 14,528 people died in institutions, representing 46,9% of all deaths in France. Of those, 10,528 died in the institution where a doctor confirmed that the symptoms were associated with COVID-19, and the remaining 4,000 died in hospital and were confirmed through testing. Broken down by type of institution, the vast majority (14,214) died in accommodation establishments for the elderly (which included independent and senior residences); 281 died in facilities for people with disabilities (which also included establishments for children, institutes for the hearing and vision impaired, as well as other establishments for adults; and 33 died in other establishments.

## Germany

In Germany, the Robert Koch-Institute publishes daily epidemiological reports on COVID-19, including mortality data on confirmed cases following a laboratory diagnosis independent of clinical assessment. By September 23, 3,690 or 39.2% of the total death cases in the whole country occurred in institutions, which include facilities for the care of older, disabled, or other persons in need of care, homeless shelters, community facilities for asylum- seekers, repatriates and refugees as well as other mass accommodation and prisons. Data are reported as a whole and not disaggregated by facility type. As the data include communal settings such as homeless shelters, homes for refugees and prisons, where younger population might also be represented, it is important to note that the information is not directly comparable with the data on care homes from other countries in this report. However, these data suggest that in Germany care residents represent a smaller share of all deaths compared to other countries with similar number of deaths in total.

## Honk Kong

No deaths in institutions have been reported by the Government of Honk Kong so far.

## Hungary

Government of Hungary reported on June 2 that 127 people died in long-term care facilities, constituting 24% of all deaths in Hungary at that date. COVID-19 deaths were defined as people who have tested positive and died.

## Republic of Ireland

At a National Public Health Emergency Team press briefing held at the Department of Health of Republic of Ireland on June 22, it was revealed that there has been a total of 1,086 COVID-19 related deaths linked to long-term care facilities, 963 of which are linked to nursing homes. These numbers represent 63.2% and 56% of all deaths that have occurred in Ireland, respectively.

## Israel

According to an internal report from the Ministry of Health of Israel published on June 24 and cited by the ILTCPN, by that date 307 deaths have been reported in the country, 137 (45%) of which have occurred in long-term care facilities. No new data have been made available since then.

## Italy

Istituto Superiore di Sanità in Italy published a report with data on confirmed and suspected deaths from COVID-19 in nursing homes for the period February 1 - May 5 2020. The report was based on a survey sent to 3,276 nursing homes out of 4,629 operating on national territory. As of May 5, based on responses from 1,356 nursing homes, the COVID-19 related mortality rate is 12,9%, including 680 confirmed and 3092 suspected deaths out of the 29,079 total deaths in the country. No new data have been made available since May 5.

## Jordan

No deaths in institutions have been reported by the Government of Jordan so far.

## Malta

No deaths in institutions have been reported by the Government of Malta so far.

## Netherlands

The Government of the Netherlands updates the number of deaths occurring in nursing homes on a daily basis. As of September 23, 2020, there were 2884 deaths that occurred in these institutions, accounting for 45,8% of all deaths in the Netherlands.

## New Zealand

According to a report published on June 26 by the ILTCPN, 16 people had died in aged residential care facilities, accounting for 72.2% % of all COVID-19 related fatalities in the country.

## Northern Ireland

The Northern Ireland Statistics and Research Agency publishes weekly epidemiological reports, including information on mortality in care homes where COVID-19 (suspected or confirmed) is mentioned on the death certificate of deceased. More specifically, the data includes deaths of care home residents, where either (a) the death occurred in a care home, or (b) the death occurred elsewhere but the place of usual residence of the deceased was recorded as a care home. In this case, the statistics does not capture those cases where a care home resident died in hospital or another location and the usual address recorded on their death certificate has not been indicated as a care home. By September 23, there have been 433 deaths of care home residents, which is 48.8% of all COVID-19 related deaths in Northern Ireland.

## Norway

The Norwegian Institute of Public Health publishes data on the number of deaths linked to COVID-19 that have occurred in institutions/care homes on a weekly basis. The report from September 23 shows that 156 confirmed deaths have occurred in care homes and other institutions, constituting 58% of all deaths in Norway.

## Portugal

The Government of Portugal released on May 9 data on the number of deaths in nursing homes, reporting 450 deaths in nursing homes, which account for 40% of all deaths in the country. No new data have been published since then.

## Scotland

National Records of Scotland publishes weekly reports on death registrations which mention COVID-19 in the death certificate, using the emergency ICD10 codes. The reports also reflect the number of deaths occurring in care homes. As of September 16, 1966 (46.4%) of the total deaths in Scotland occurred in care homes.

## Singapore

The Ministry of Health of Singapore publishes daily epidemiological reports on their webpage. As of August 3, there have been 27 total confirmed deaths in the country, 2 of which have occurred in nursing homes, accounting for 7.4% of the total mortality.

## Slovenia

The Government of Slovenia announced on May 20 that until that date, out of 105 deaths in the country, 52% happened in care homes and another 29% were deaths of care home residents that happened in a hospital. This brings the total proportion of deaths of care home residents to 81%.

## South Korea

The ILTCPN published a report showing that by April 20, out of 247 deaths linked to COVID-19 in South Korea, 20 (8.1%) were people who have been infected in nursing homes, and additional 64 (25.9%) who died in long-term care hospitals. This makes the total share of deaths in institutions 34% of total deaths. It has to be noted that no death occurred in an institution as all COVID-19 cases were transferred to hospitals.

## Spain

Official data from regional governments in Spain show that as of September 23, a total of 20,237 deaths have occurred in long-term care facilities for older people, accounting for 65.2% of all deaths in the country. This number includes both people who have been diagnosed with COVID-19 and those who have shown symptoms of the illness but have not been tested.

## Sweden

The National Board of Health and Welfare of Sweden publishes weekly epidemiological reports including information on place of death (hospital or care home). As of September 23, 2627 people had died in assisted care homes which constitutes 45.2% of all deaths in the country.

## Switzerland

Cantonal data collected by the independent Tages-Anzieger newspaper shows that by May 19, a total of 53% of the country’s 1891 deaths had occurred in nursing homes. It has to be noted that these figures come from just 18 of the country’s 26 cantons. However, the deaths in these 18 cantons accounted for 94% of Switzerland’s total coronavirus deaths by the time the analysis was undertaken. No updates have been published since then.

## Turkey

According to an ILTCPN report, as of May 7, 150 deaths of care home residents have been reported, accounting for 4% of all COVID-19 deaths in Turkey. These data need to be seen with caution as no official government information has been published.

## Wales

In Wales, the Office for National Statistics (ONS) provides weekly updates of registered deaths which differ from the figures provided by NHS in that they include all deaths where “COVID-19” was mentioned (by a doctor) on death certificates (using ICD10 codes U07.1 and U07.2). Up to September 11, there were 706 deaths that occurred in care homes in England – a 27,5% of all COVID-19 related deaths in the country. Care homes include homes for the chronic sick, nursing homes, homes for people with mental health problems and non-NHS multi-function sites. In addition, 15 deaths occurred in hospices, which include Sue Ryder Homes, Marie Curie Centres, oncology centres, voluntary hospice units, and palliative care centres. Another 15 deaths occurred in other communal establishments including schools for people with learning disabilities, holiday homes and hotels, common lodging houses, aged persons’ accommodation, assessment centres, schools, convents and monasteries, and nurses’ homes.

## United States

Kaiser Family Foundation publishes updated epidemiological information on COVID-19 related mortality in long-term care homes. As of September 23, 80,006 deaths have occurred in care homes accounting for 40% of all deaths in the United States.

##

## Methodology and Search Strategy

Data were searched for the period February 1 – September 23, 2020. There were five main sources of information: PubMed for peer reviewed scientific literature, Google for free search for grey literature, published reports from the International Long-Term Care Policy Network (ILTCPN), the Epidemic Intelligence from Open Sources (EIOS) platform for media monitoring, and Sprinklr for social media monitoring.

A search was performed in PubMed for any scientific literature that contains data on COVID-19 related mortality in institutions. The following MeSH terms and keywords were combined in a search string: *coronavirus OR COVID-19 or SARS-CoV-2 AND care home OR institution* OR nursing home OR retirement home OR homes for the aged OR nursing facility OR residential home OR long term care OR long-term care AND death* OR mortality.* A researcher undertook the initial screening of titles/abstracts and the following full texts of selected articles. Selection was double checked for consistency by a second researcher.

Search for grey literature was performed in Google to capture any published sources of information, including governmental websites, new reports, professional organizations’ databases, epidemiological reports, social media messages, etc. In addition, the reports published by ILTCPN were checked and information was extracted.

A detailed search was also performed in the EIOS platform. EIOS is a collaboration between WHO and various stakeholders that provides a web-based system for monitoring and early detection (<https://www.who.int/eios>). The system combines existing systems including the Early Alerting and Reporting system (Global Health Security Initiative) and the Hazard Detection and Risk Assessment System (WHO), both developed by the European Commission’s Joint Research Centre (JRC). On a daily basis, the system collates thousands of articles from a broad range of sources such as government and official sites, specific social media sources, news aggregators, and expert groups. Through series of text mining and analytical modules the platform runs this information to sort and categorize it by category, country, language, and source. The platform then allows users to browse, search, filter, read, and export articles.

To ensure comprehensiveness in the search, we used the platform in two ways. On one hand, we searched across all COVID-19 related articles available in the platform through a keyword Boolean string which combined terms indicative of mortality and institutions. This free-text search provides the advantage of looking at sources retrospectively and automatically obtain results in different languages. However, the disadvantage of this method is that the search needs to be updated manually on a daily basis to retrieve newly published articles. Therefore, we also created a predefined “category” – a tool available in EIOS, which automatically allows the system to detect and classify articles related to a topic of interest as they appear in the media. The created category was based on a keyword pattern indicative of COVID-19 mortality in institutions and used terms translated in the six official UN languages – English, Arabic, Spanish, Russian, French and Chinese. The category was specifically designed to catch future articles coming into the system. The advantage of this method is that it provides an automatic update on a daily basis, but the disadvantage is that articles published before its creation cannot be identified.

The last source of information was Sprinklr - a social media management tool that helps undertake research on social media channels such as Facebook, Twitter and 21 others- all in one integrated platform (<https://www.sprinklr.com/>). Social media messages were checked if they contained links or information which could be traced to its source. Key messages indicative of COVID-19, mortality and institutions were used for the search.

The identified sources were checked for any data disaggregation on disability such as age and gender differences or type of facilities (e.g. nursing homes, psychiatric units, prisons, etc).

## References

1. Government of Australia: [https://www.health.gov.au/news/health-alerts/novel-coronavirus-2019-ncov-health-alert/coronavirus-COVID-19-current-situation-and-case-numbers](https://www.health.gov.au/news/health-alerts/novel-coronavirus-2019-ncov-health-alert/coronavirus-covid-19-current-situation-and-case-numbers)
2. Government of Austria: <https://goeg.at/PK_COVID-19_in_Alten-_und_Pflegeheimen>
3. Sciensano. (2020). COVID-19 – Bulletin Epidemiologique du 7 Julliet 2020. Available at: [https://COVID-19.sciensano.be/sites/default/files/Covid19/Derni%C3%A8re%20mise%20%C3%A0%20jour%20de%20la%20situation%20%C3%A9pid%C3%A9miologique.pdf](https://covid-19.sciensano.be/sites/default/files/Covid19/Derni%C3%A8re%20mise%20%C3%A0%20jour%20de%20la%20situation%20%C3%A9pid%C3%A9miologique.pdf)
4. Hsu AT, Lane NE, Sinha SK, Dunning J, Dhuper M, Kahiel Z, Sveistrup H. Report: Understanding the impact of COVID-19 on residents of Canada’s long-term care homes — ongoing challenges and policy responses. LTCcovid, International Long-Term Care Policy Network, CPEC-LSE. Available at: <https://ltccovid.org/wpcontent/uploads/2020/05/LTCcovid-country-reports_Canada_Hsu-et-al_May-10-2020-2.pdf>
5. <https://www.ssi.dk/sygdomme-beredskab-og-forskning/sygdomsovervaagning/c/covid19-overvaagning>
6. Office for National Statistics of England and Wales: <https://www.ons.gov.uk/peoplepopulationandcommunity/birthsdeathsandmarriages/deaths/datasets/weeklyprovisionalfiguresondeathsregisteredinenglandandwales>
7. Finnish Institute for Health and Welfare: [https://thl.fi/en/web/infectious-diseases-and-vaccinations/what-s-new/coronavirus-COVID-19-latest-updates/situation-update-on-coronavirus](https://thl.fi/en/web/infectious-diseases-and-vaccinations/what-s-new/coronavirus-covid-19-latest-updates/situation-update-on-coronavirus)
8. Government of France: [https://www.santepubliquefrance.fr/maladies-et-traumatismes/maladies-et-infections-respiratoires/infection-a-coronavirus/documents/bulletin-national/COVID-19-point-epidemiologique-du-2-juillet-2020](https://www.santepubliquefrance.fr/maladies-et-traumatismes/maladies-et-infections-respiratoires/infection-a-coronavirus/documents/bulletin-national/covid-19-point-epidemiologique-du-2-juillet-2020)
9. Robert Koch Institute. (2020). COVID-19 daily situation report: 7 July. Available at: <https://www.rki.de/DE/Content/InfAZ/N/Neuartiges_Coronavirus/Situationsberichte/2020-07-07-en.pdf?__blob=publicationFile>
10. Government of Hong Kong: [https://chp-dashboard.geodata.gov.hk/COVID-19/en.html](https://chp-dashboard.geodata.gov.hk/covid-19/en.html)
11. Government of Hungary: <https://koronavirus.gov.hu/cikkek/az-idosotthonokban-fertozottek-fele-mar-meggyogyult>
12. Republic of Ireland Department of Health. (2020). COVID-19 Press Briefing: Available at: <https://twitter.com/roinnslainte/status/1275107587810263041>
13. Government of Israel: <https://www.gov.il/he/departments/publications/reports/daily-report-07072020>
14. Comas-Herrera A, Zalakaín J, Litwin C, Hsu AT, Lemmon E, Henderson D and Fernández J-L (2020) Mortality associated with COVID-19 outbreaks in care homes: early international evidence. Article in LTCcovid.org, International Long-Term Care Policy Network, CPEC-LSE, 26 June 2020.
15. National Health Institute of Italy: <https://www.epicentro.iss.it/coronavirus/pdf/sars-cov-2-survey-rsa-rapporto-finale.pdf>
16. Government of Malta: [https://deputyprimeminister.gov.mt/en/health-promotion/COVID-19/Pages/COVID-19-infographics.aspx](https://deputyprimeminister.gov.mt/en/health-promotion/covid-19/Pages/covid-19-infographics.aspx)
17. Government of The Netherlands: <https://coronadashboard.rijksoverheid.nl/>
18. Government of New Zealand: [https://www.health.govt.nz/our-work/diseases-and-conditions/COVID-19-novel-coronavirus/COVID-19-current-situation/COVID-19-current-cases](https://www.health.govt.nz/our-work/diseases-and-conditions/covid-19-novel-coronavirus/covid-19-current-situation/covid-19-current-cases)
19. Northern Ireland Statistics and research Agency: <https://www.nisra.gov.uk/publications/weekly-deaths>
20. <https://www.fhi.no/contentassets/8a971e7b0a3c4a06bdbf381ab52e6157/vedlegg/andre-halvar-2020/2020.09.23-ukerapport-uke-38-covid-19.pdf>
21. <https://www.sabado.pt/portugal/amp/coronavirus-ja-morreram-450-idosos-em-lares>
22. National Records of Scotland: [https://www.nrscotland.gov.uk/statistics-and-data/statistics/statistics-by-theme/vital-events/general-publications/weekly-and-monthly-data-on-births-and-deaths/deaths-involving-coronavirus-COVID-19-in-scotland/related-statistics](https://www.nrscotland.gov.uk/statistics-and-data/statistics/statistics-by-theme/vital-events/general-publications/weekly-and-monthly-data-on-births-and-deaths/deaths-involving-coronavirus-covid-19-in-scotland/related-statistics)
23. Ministry of Health of Singapore: <https://www.moh.gov.sg/>
24. <https://www.gov.si/novice/2020-05-29-celotna-izjava-dr-beovic-glede-zdravstvene-oskrbe-stanovalcev-domov-za-starejse/>
25. [https://china-cee.eu/2020/06/15/slovenia-social-briefing-COVID-19-in-nursing-homes-and-the-controversy-about-official-guidelines/](https://china-cee.eu/2020/06/15/slovenia-social-briefing-covid-19-in-nursing-homes-and-the-controversy-about-official-guidelines/)
26. <https://www.rtve.es/noticias/20200706/radiografia-del-coronavirus-residencias-ancianos-espana/2011609.shtml>
27. Government of Spain: <https://www.mscbs.gob.es/profesionales/saludPublica/ccayes/alertasActual/nCov-China/home.htm>
28. National Board of Health and Welfare: [https://www.socialstyrelsen.se/statistik-och-data/statistik/statistik-om-COVID-19/statistik-over-antal-avlidna-i-COVID-19/](https://www.socialstyrelsen.se/statistik-och-data/statistik/statistik-om-covid-19/statistik-over-antal-avlidna-i-covid-19/)
29. <https://interaktiv.tagesanzeiger.ch/2020/corona-tote-mehrheitlich-aus-altersheimen/>
30. [https://www.kff.org/coronavirus-COVID-19/issue-brief/state-data-and-policy-actions-to-address-coronavirus/](https://www.kff.org/coronavirus-covid-19/issue-brief/state-data-and-policy-actions-to-address-coronavirus/)
